# Hygiene measures and antimicrobial use practices in households of a rural community in South India: a cross-sectional study

**DOI:** 10.3389/fpubh.2025.1753160

**Published:** 2026-01-09

**Authors:** Philip Mathew, Sujith John Chandy, Cecilia Stålsby Lundborg

**Affiliations:** 1 Department of Global Public Health, Karolinska Institutet, Stockholm, Sweden; 2 Department of Pharmacology and Clinical Pharmacology, Christian Medical College, Vellore, India

**Keywords:** antibiotic use, antimicrobial resistance, behavior change, community engagement, hygiene, infection prevention

## Abstract

**Background:**

Inadequate water, sanitation and hygiene (WASH) practices in community settings is a driver for antimicrobial use and antimicrobial resistance (AMR). Understanding the WASH and antimicrobial use practices in communities, can potentially open opportunities to enhance AMR awareness and stimulate action.

**Methods:**

A cross sectional study was conducted in 400 households the state of Kerala, India. Basic socio demographic details of the household, antimicrobial use practices of the household and data on WASH practices and provisions were collected, using a pilot-tested questionnaire.

**Results:**

Sixteen percent of the households reported use of antimicrobials at least once in the last 12 months and all obtained it using valid prescriptions. The commonest reason cited for the use of antimicrobials was fever (33.8%), upper respiratory infections (17.7%) and urinary tract infection (12.9%). Access to piped drinking water and improved sanitation was universal in the area. Solid waste management was a challenge with most households (384, 96%) practicing open dumping. Larger household size and presence of children in the household were factors influencing antimicrobial use.

**Discussion:**

The household level antimicrobial use was lower than other estimates from similar contexts and all the antimicrobials were obtained with valid prescriptions. This may be partially due to stricter implementation of regulations in the state. The optimal access to safe drinking water and improved sanitation facilities in the area may be responsible for the low use of antimicrobials in the households. Therefore, WASH should be an integral part of the action plans on AMR, especially those in sub-national settings.

## Introduction

Antimicrobial Resistance (AMR) is an existential threat to modern medicine. The number of deaths associated with AMR is estimated to be around 4.95 million per year in 2019, out of which 1.27 million can be directly attributed to it (1). It is estimated cause 39 million deaths between 2025 and 2050, with directly attributable deaths rising to 1.91 million annually by 2050 (2). This is significantly higher than previous estimates and makes AMR a bigger problem than Human Immunodeficiency Virus (HIV) infection or Malaria (3). Besides mortality, it also increases morbidity through longer hospital stays, higher likelihood of admission to Intensive Care Units and more instances of treatment failures (4). The cost of treating infections with multi-drug resistant bacteria is also significantly higher, when compared to susceptible microorganisms (5). There is an economic cost to AMR, primarily due to the loss of productivity associated with morbidity and premature mortality and the cost of healthcare. The World Bank estimates that AMR will reduce global Gross Domestic Product (GDP) between 1.1 and 3.7%, after 2030 (6). The issue is driven by inappropriate antimicrobial use in multiple sectors- human healthcare, food animal production, horticulture and aquaculture. Environment often plays the role of a disseminator and amplifier of resistance, as most of the antimicrobials used in various sectors end up in the environment (7). Therefore, AMR is truly a One-Health problem and warrants concerted action from various sectors to effectively mitigate it.

The emergence and spread of AMR is closely linked to drinking water, hygiene and sanitation. Poor access to clean drinking water and proper sanitation can increase the likelihood of infections in the community, which increases the requirement of antimicrobials. Inadequate sanitation can also result in antimicrobial residues, antimicrobial resistance genes and resistant microorganisms interacting with each other in the environment, with a higher risk of novel AMR pathogens emerging and spreading (8). Improper waste management in farms, poor quality infection prevention & control (IPC) in healthcare delivery sites and sub-optimal sewage treatment are other interfaces between AMR and hygiene (9). Therefore, inadequate water, sanitation and hygiene (WASH) practices and provisions influences AMR in all associated sectors- human health, animal health, agriculture and environment. Improving hygiene and sanitation practices becomes important in the fight against AMR and has been acknowledged in the Global Action Plan on Antimicrobial Resistance (GAP-AMR) published by World Health Organization (WHO) (10).

There are several calls for greater community engagement on AMR, acknowledging that the current narratives of AMR action plans are mostly top down and there is a need to complement them with bottom-up approaches (11). Since AMR is largely an invisible issue and the framing is complex, the communities find it difficult to prioritize AMR (12), and hygiene can be used as a theme to create interest and drive the AMR agenda in local contexts, especially in Low and Middle Income Country (LMIC) contexts (13). If the linkage between AMR and WASH is clearly evident to the public and local level decision makers, they may be motivated to act on it. Hygiene can also be used as an entry point for awareness-raising and public engagement on AMR issues. But there are no standardized sources of information for in-depth information on household level hygiene and antimicrobial use practices in countries like India. Therefore, a multi-dimensional assessment of drinking water, hygiene and sanitation practices in communities, using an AMR-lens, was conducted in a rural community in south India to understand the hygiene practices, and its linkage, if any, with antimicrobial use practices.

## Methods

### Study design and setting

**Figure 1:**
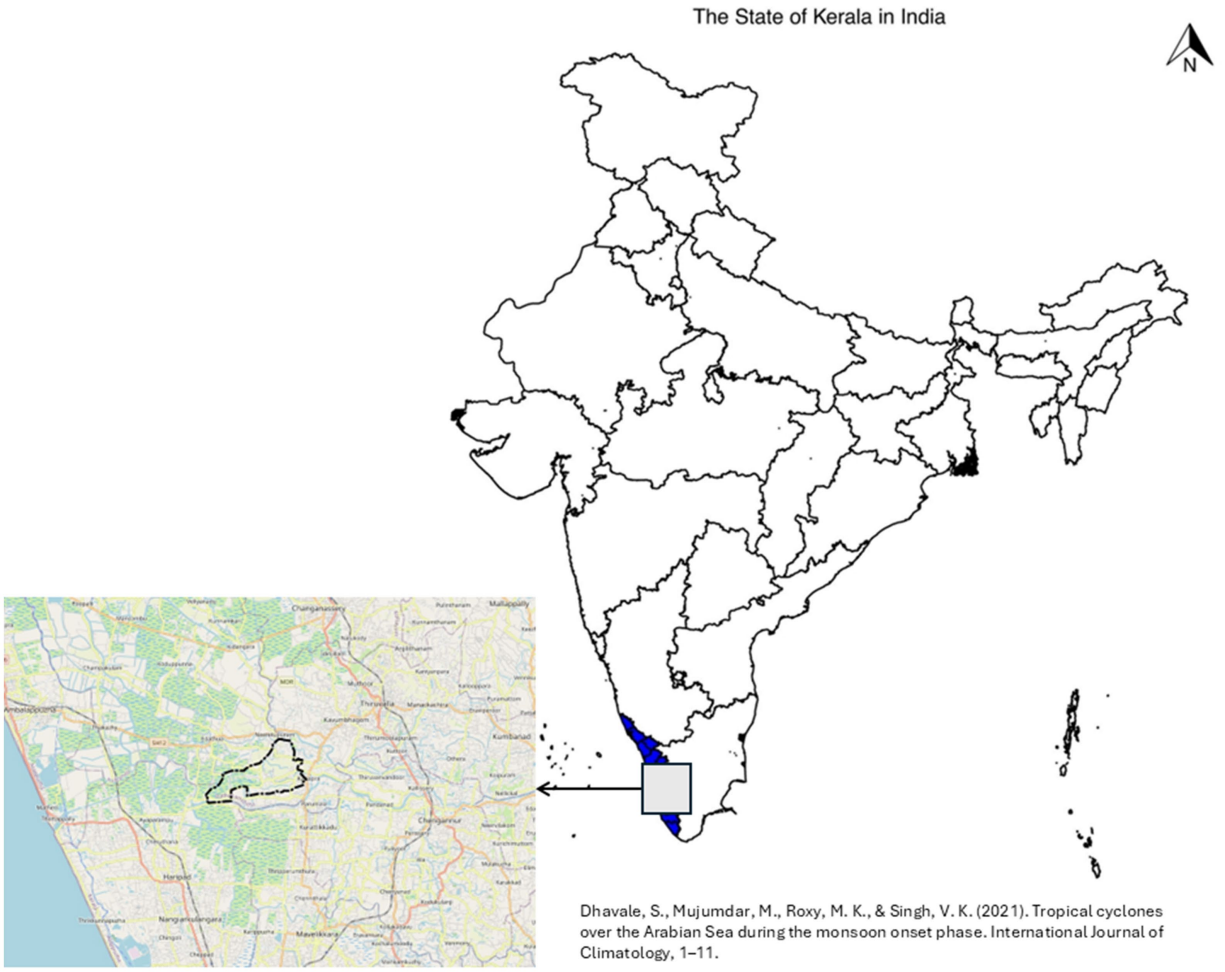
Location of the study site–Niranam *panchayat*, Kerala, India. Source: OpenDataKerala.

A cross-sectional study was conducted in the area of a local self-government institution in the state of Kerala, India (Figure 1). Local self-governance in India is led by a multi-tiered structure, which has relatively limited functional autonomy. These institutions have a significant influence on delivery of primary health, animal health, agriculture and WASH services by the state and national governments (14). Kerala is the first state to have a state action plan on AMR in the country (15). The state has a literacy rate of 94%, a sex ratio of 1084 females per 1000 males and a high Human Development Index (HDI) of 0.758 (16). The state is known for a robust democratic decentralization process, which has empowered local self-government institutions to actively intervene in health, hygiene and social protection (17). The survey was conducted in Niranam, a *panchayat* (a local administrative structure which vary from states to state), during 2022–2023. Niranam was selected for the study because of high-level of access to households, and the interest shown by the local self-government institution. The demography, literacy and vital statistics of Niranam are comparable to the rest of the state. The *panchayat* selected for the study had a population of 14,774 spread over 13.17 sq.kms, as per the last available census data. The *panchayat* is a part of the Kuttanad region, which is mostly below the mean sea level with extensive land reclamation in the last century. Rice farming is a thriving sector in the region, but the area under cultivation has consistently fallen in the last few decades. The low-lying terrain and the prevalence of rice paddy fields make the panchayat prone for water logging. The seasonal flooding also reduces the water quality, with high probability of fecal matter contamination because of septic tanks getting clogged (18).

### Data collection instrument

The survey questionnaire was adapted from Eurobarometer 445 which was published by the European Commission for conducting country level surveys on use of antimicrobials (19), *Swachh Sarvekshan Grameen* (commissioned by the government of India to rank villages on hygiene) (20) and Core questions on Drinking Water & Sanitation (published by WHO & UNICEF) (21). These sources were used since the tools are validated and the questions are widely adopted. The tool was reviewed by four experts in the topic before being finalized. The tool was translated to the local language by the investigators and back translated by an independent expert, to ensure validity. The tool was pilot tested among selected households before being used widely for data collection.

### Sample size and sampling strategy

Household was the unit of sampling for the study. Four hundred households, out of approximately 3500 households in the *panchayat*, were included in the study, based on an assumption that 50% of households follow proper hygiene practices, along with an alpha error of 5% and a relative precision of 10%. A modified systematic random sampling methodology was followed for the study. Two habitations were randomly selected from each of the 14 wards of the panchayat. In each habitation, one direction was randomly selected from the centre of the habitation and a number (n) was randomly chosen between 1 and 10. Every n^th^ house was selected for the study. Households with medical professionals or qualified pharmacists were excluded from the study. None of the selected households denied consent for the study.

### Data collection methods

The households were approached through the community health extension workers (ASHAs) and the interviews were conducted in the local language Malayalam, at the premises of the houses. The head of the family was selected for the interview, if available at the time of the visit. Otherwise, the oldest cognitively stable member of the household was interviewed by a trained health worker. This was done to ensure that high-quality information is elicited during the interview. For the study, the Indian joint family was considered as those having “three to four living generations, including grandparents, parents, uncles, aunts, nieces and nephews, all living together in the same household, utilizing a common kitchen and often spending from a common purse, contributed by all” (22). Short, structured information on antimicrobials was given and a few samples of commonly used antimicrobials in the area were shown to the participants before the interview. This was done with an assumption that most respondents may not be able to differentiate between antimicrobials and other medicines, which can lead to significant information bias. Global Positioning System (GPS) coordinates of the house was mapped for the intervention proposed later. Basic socio demographic details of the household, antimicrobial use practices of the household and data on WASH practices and provisions were collected.

### Data management and analysis

Field data entry was done using Kobo Toolbox. The data was analysed using SPSS 23.0. The proportion of households which had sub-optimal hygiene and antimicrobial use practices was assessed and expressed as frequency/percentages. A 95% confidence interval was calculated for all the proportions. The association of antimicrobial use practices with baseline household variables was assessed using Chi-Square test. Odds ratios (95% Confidence interval) were calculated wherever needed, to evaluate the strength of association. Binary logistic regression was done to look at factors predicting antimicrobial use practices in households.

### Ethical considerations

Ethics clearance (PIMS/IRB/03/2019) was given by the Institutional Review Board (IRB) of Pushpagiri Institute of Medical Sciences and Research Centre, Thiruvalla, Kerala, India. Written Informed Consent in local language was collected from all the respondents before data collection, after explaining the objectives and process of the study. Internationally accepted norms of data safety and confidentiality were followed throughout the study along with all relevant aspects of the Declaration of Helsinki 1964 (23).

## Results

The study was conducted in 2022–23. Most (258, 65%) of the informants were females and all of them were literate. A vast majority of them (83%) were not educated beyond high school, with almost 60% of them not being a part of the labor market. Regarding the households which were included in the study, almost all (393, 98%) of the participants owned their own houses or land. A significant number (136, 34%) of respondents mentioned that their households had three-generations, but none were joint families (Table 1).

**Table 1:** Characteristics of the households and informants in the study.

Characteristics	*n* (%)
Informants (*N* = 400)	
Sex	
Male	142 (35)
Female	258 (65)
Education	
Professional degree	2 (0.5)
Graduate or Postgraduate	27 (7)
Intermediate or post-high school diploma	39 (10)
High school	145 (36)
Middle school	186 (47)
Primary school	1 (0.3)
Illiterate	0
Occupation	
Professional occupations	1 (0.3)
Semi-Professional occupations	14 (4)
Clerical work, shop owner, farmer	39 (10)
Skilled worker	26 (7)
Semi-skilled worker	10 (3)
Unskilled worker	56 (14)
Unemployed	239 (60)
Student	15(4)
Family	
Education of head of family	
Professional degree	3 (0.8)
Graduate or Postgraduate	24 (6)
Intermediate or post-high school diploma	31 (8)
High school certificate	155 (39)
Middle School Certificate	184 (46)
Primary School Certificate	3 (0.8)
Illiterate	0
Occupation of head of family	
Professional occupations	3 (0.8)
Semi-Professional occupations	20 (5)
Clerical work, shop owner, farmer	74 (19)
Skilled worker	71 (18)
Semi-skilled worker	14 (4)
Unskilled worker	148 (37)
Unemployed	70 (18)
Land ownership	
Yes	393 (98)
No	7 (2)
Subsistence farming	
Yes	137 (34)
No	263 (66)
Type of family	
Nuclear	264 (66)
Three-generation	136 (34)
Joint	0
Household Size	
4 and above	201 (50)
Up to 3	199 (50)
Household animals	
Yes	80 (20)
No	320 (80)

Only 15.5% of the households reported use of antimicrobials at least once in the last 12 months and they reported that they had obtained it using prescriptions from medical practitioners. The commonest reason cited for the use of antimicrobials was fever (33.8%), followed by upper respiratory infections (17.7%) and urinary tract infection (12.9%). We did not include the doxycycline prophylaxis for leptospirosis, albendazole prophylaxis for helminthiasis and the diethylcarbamazine prophylaxis for Filariasis in these statistics, as it is almost universally given in the area by the health department of the state government. Only 4 (5%) of the animal keeping households reported antimicrobial use, and the medicines were reported to have been obtained with a prescription in all of the instances (Table 2).

**Table 2:** Antimicrobial use practices in the community.

Practices and choices	*n* (%)
Antimicrobial use in humans	
Households which reported use of antimicrobials at least once in the last 12 months	
Yes	62 (16)
No	338 (85)
If antimicrobials used, how did they obtain it?	
Prescription from a medical practitioner	62 (100)
Over-the-counter, without prescription	0
Others	0
If used antimicrobials, why was it used?	
Fever	21 (34)
Cough/Cold	11 (18)
Urinary Infection	8 (13)
Wounds	7 (11)
Skin Infection	4 (7)
Others	10 (16)
If used antimicrobials, when was it stopped	
On symptom relief	61 (98)
On completing the course	1 (1.6)
On starting other antimicrobials	0
Other reasons	0
When getting antimicrobials, did they receive information on using and stopping	
Yes	62 (100)
No	0
Experienced any adverse reaction associated with antimicrobial use	
Yes	0
No	62 (100)
Antimicrobial use in animals (*N* = 80)	
Households reporting antimicrobials use in animals, among all animal-keeping households	
Yes	4 (5)
No	76 (95)
If used antimicrobials, how did they obtain it?	
Prescription from a veterinary practitioner	4 (100)
Over-the-counter, without prescription	0
Others	0
If used antimicrobials, why was it used?	
Treatment of infection	2 (50)
Prevention of disease	2 (50)
Others	0

The households were dependent on piped water and protected wells for their drinking water needs; and availability of water was not an issue. Access to proper sanitation was also universal in the area, with all the households having flush/pour toilets. Soap for handwashing was also available universally in the toilets in the households and there was good usage of the hand washing facilities. The area was well covered by teams engaged for collection, segregation and disposal of waste; and most (396, 99%) households use the services. Since they do not collect organic waste, households (384, 96%) practice open dumping in their yards. All the households dispose medicines including antimicrobials, using the same methods as general household waste (Table 3).

**Table 3:** Water, hygiene and sanitation (WASH) practices in the community.

Practices and choices	*n* (%)
Water	
Main sources of drinking water^#^	
Piped water	218 (54.5)
Protected well	256 (64.0)
Tube well	8 (2.0)
Delivered water	14 (3.5)
Main source of water for household purposes^#^	
Piped water	278 (69.5)
Protected well	376 (94.0)
Tube well	5 (1.3)
Delivered water	2 (0.5)
Availability of drinking water at the primary source^#^	
Always	365 (89.0)
Most of the time	1 (0.3)
Sometimes	43 (10.8)
Storage of drinking water before consumption in households^#^	
Steel containers	392 (98.0)
Earthen vessels	32 (8.0)
Plastic containers	2 (0.5)
Disinfection of drinking water before consumption^#^	
Boiling	400 (100)
Water filters	1 (0.3)
Chlorine tablets	0
Other	0
Sanitation and waste disposal	
Access to improved sanitation	
Toilets within the premises of the house	400 (100)
Shared or community toilets	0
No access to toilets	0
If toilet present in the premises of the house, type	
Flush/pour toilet connected to septic tank	399 (99.7)
Flush/pour toilet connected to pit latrine	1 (0.3)
Flush/pour toilet connected to sewerage system	0
Bore hole latrine	0
Availability of soap in the toilets for hand washing	
Soap bar	392 (98.0)
Liquid Soap	1 (0.3)
Both	7 (1.7)
No soap	0
Family members washing hands after using toilets	
Always	399 (99.7)
Most of the times	1 (0.3)
Some of the times	0
Never	0
Methods for disposing household waste^#^	
Use formal service provider who do collection	396 (99.0)
Dispose in household yard	384 (96.0)
Designated areas in the village	2 (0.5)
Open dumping	0
Composting	0
Methods for disposing household waste water	
Drains connected to soakage pits/pits	397 (99.2)
Drains connected to septic tank	3 (0.8)
Surface run-off	0
Methods for disposing unused or expired medicines, including antimicrobials	
Along with household garbage	400 (100)
Returned to pharmacies	0
Specialized disposal	0
Hand-washing facilities inside the house^#^	
Tap and Sink	400 (100)
Pour-type	18 (4.5)
No facility inside the house	0
Availability of soap for hand-washing	
Soap bar	395 (98.8)
Liquid Soap	5 (1.2)
No soap available	0
Adherence of family members to hand washing before meals	
Always before meals	400 (100)
Most of the times before meds	0
Sometimes before meals	0
Never	0

^#^Totals are not equal to sample size because of multiple responses by the same respondents.

According to the survey, only 62 (15.5%) of the households had consumed antimicrobials for any indication in the preceding 12 months. Some of the baseline characteristics of the household were selected for tests of association, based on plausibility and reports in literature. Out of the characteristics which were tested, household size of 4 or more (*p* < 0.001, OR 3.39, 95% CI- 1.84 to 6.23) and presence of children in the household (*p* < 0.001, OR- 2.57, 95% CI- 1.46 to 4.51) increased the risk of antimicrobial consumption, while poor occupational status of the head of the family (*p*- 0.026, OR- 0.54, 95% CI- 0.31 to 0.93) decreased the risk (Table 4).

**Table 4:** Association of antimicrobial consumption in the last 12 months with baseline characteristics of the household.

Characteristics	Households with antimicrobial consumption	Households without antimicrobial consumption	*p* value	OR (95% CI)
Household size			<0.001^@^	3.39 (1.84 to 6.23)
4 or above	46 (22.9%)	155 (77.1%)		
Upto 3	16 (8.0%)	183 (92.0%)		
Children in the household			<0.001^@^	2.57 (1.46 to 4.51)
Children present	40 (22.2%)	140 (77.8%)		
No children	22 (10.0%)	198 (90.0%)		
Earning members			0.349	1. 31 (0.74 to 2.30)
More than 1	23 (18%)	105 (82%)		
Upto 1	39 (14.3%)	233 (85.7%)		
Education of the head of the family			0.612	0.86 (0.50 to 1.49)
Upto Middle School	27 (14.5%)	159 (85.5%)		
High School and above	35 (16.4%)	179 (83.6%)		
Occupation of the head of the family			0.026^@^	0.54 (0. 31 to 0.93)
Unemployed up to semi-skilled	28 (12.1%)	204 (87.9%)		
Skilled and above	34 (20.2%)	134 (79.8%)		
Primary healthcare provider			0.075	1.66 (0.95 to 2.93)
Private	24 (20.5%)	93 (79.5%)		
Government	38 (13.4%)	245 (86.6%)		

@ *p* < 0.05 was taken as statistically significant.

A binary logistic regression was done to account for confounding variables, since many of the baseline characteristics of the household are closely linked to each other. In the regression model, only household size (*p*- 0.012, Adjusted OR- 0.367, 95% CI- 0.167 to 0.804) was significantly associated with antimicrobial use in the household (Table 5).

**Table 5:** Binary logistic regression model for antimicrobial consumption in the household in the last 12 months.

Variables	B	Std Error	Wald	*p* value	Adjusted OR (95% CI)
Household size	−1.004	0.401	6.268	0.012	0.367 (0.167 to 0.804)
Children in the household	−0.348	0.361	0.927	0.336	0.706 (0.348 to 1.433)
Earning members	0.153	0.318	0.231	0.630	1.165 (0.625 to 2.172)
Education of the head of the family	−0.120	0.300	0.160	0.689	0.887 (0.493 to 1.596)
Occupation of the head of the family	0.285	0.336	0.720	0.396	1.330 (0.688 to 2.571)
Primary healthcare provider	−0.222	0.344	0.417	0.518	0.801 (0.408 to 1.571)
Source of drinking water	−0.354	0.310	1.302	0.254	0.702 (0.382 to 1.290)
Constant	2.651	0.457	33.62	<0.001	

## Discussion

The proportion of households reporting antimicrobial use is significantly lower than other estimates (24) from India, though the period of recall has been different. A similar study from Bangladesh reported that 70% of the population has previously taken antibiotics, with 21% reporting use within the last one month of the survey (25). Household level antimicrobial use has been reported to be significantly higher, from other low and middle income countries like Ghana (26), Malawi (27) and Kenya (28), even though the socio-economic indices may be different from that of Kerala, India. A study done in Burkina Faso showed that 49% of the households procured and stored antibiotics, for it to be used as self-medication for several health issues (29). Since the survey was conducted after the Covid19-induced lockdowns of 2020 and 2021, this may have reflected in the antimicrobial use statistics. Though there are several studies showing inappropriate use of antimicrobials during Covid19, the lockdowns and higher adherence to hygiene measures may have had an impact on the incidence of other infections. This may have reduced the requirement of antimicrobials overall (30). Another important finding is that the antimicrobials were obtained with prescriptions from medical practitioners. Since we relied on self-reported information without any objective verification system, there may have been a conformity bias. Even then, this is significantly different from the findings reported from other parts of the country (31) and internationally from high-income contexts (32), where some or most of the antimicrobials are sold without a prescription. This may be partially due to the better regulatory system and stricter implementation of regulations that we see in the state of Kerala and also because of Schedule H1 guidelines. Schedule H1 is a list of prescription only medicines, mostly newer generation antimicrobials, anti-tuberculosis drugs and non-opioid hypnotics, published by the Government of India in 2013. This is subjected to stringent oversight, with an aim of preventing its misuse (33). Along with cultural factors unique to the state, this may have positively impacted the general awareness about antimicrobial misuse among retail pharmacists and prevented dispensing of antimicrobials without a valid prescription. The indication for antimicrobial use is similar to the findings of many other studies done in the country. Fever and upper respiratory infection syndromes are the most common reasons for antimicrobial use, especially in primary care settings (34). Since the area does not have commercial food animal farms, the use of antimicrobials in animals is also not a prominent finding.

The area had good access to clean drinking water and sanitation facilities. Households mostly depended on piped water supply and protected wells for their drinking water and household water requirements. Majority of the households (278, 69.5%) had piped water coming to their households, which is a high proportion compared to the rest of the country (35). This water supply system is maintained by the panchayat and the purification is done by chlorination only. Therefore, there can be water quality and turbidity issues during flooding, as there is no filtration system as a part of the purification process. Although 89% of the respondents mentioned that availability of drinking water is not a problem, most households store water in steel containers before consumption. Another significant aspect is that all households use boiling as a method to disinfect drinking water before consumption. These practices are significantly better than findings from the National Sample Survey done in 2017–18, showing the general understanding of the community regarding safe drinking water, and disinfection process. The frequent flooding and the subsequent inundation of the wells may also be the reason behind all households boiling the water before consumption. These optimal WASH practices may be the reason for lower household-level antimicrobial use in the community, as reported in results.

All the households had access to improved sanitation (36) and had soap to wash their hands after use of toilets. Though open defecation has been consistently decreasing after the launch of the *Swachh Bharat* programme by the Government of India, many parts of the country still have poor access to toilets (37). Launched in 2014, *Swachh Bharat* programme is a multi-billion dollar project to ensure universal access to sanitation in the country (38). Kerala has been a front-runner in eliminating unrecommended methods of disposing human waste and was declared Open Defecation Free (ODF) in 2018 (39). Since Niranam is a low-lying area with frequent flooding, it is likely that open defecation increases the likelihood of water-borne diseases. This may be the reason for prioritization of sanitation in the area. The adherence to sanitation norms may have been reinforced by the onset of Covid19 pandemic. There are adequate provisions of hand washing with soap and water inside the houses too, with high levels of compliance. The good practices related to hygiene and sanitation may also be reflective of the advanced human development indicators and high levels of social development in the state (40). Kerala has consistently performed well for almost all health indicators among bigger states in India (41).

There is an organized system for collecting and disposing household waste, led by a group of government supported self-help groups called *Haritha Karma Sena*. This group is active in the area and charges Rs. 50 (US$0.6) a month for collecting household waste. However, *Haritha Karma Sena* does not collect biodegradable waste from the houses and this creates a problem for households which do not have provision to dispose them off otherwise. Therefore, disposal in the household yard or elsewhere is practiced by 96% of the households covered through the survey. Though none of the participants of the survey indicated that they practice open dumping, there were piles of garbage seen in some side-roads in the *panchayat.* This is a serious public health concern, since this can be a driver for water-borne diseases in community settings, especially since the area is low lying and prone to frequent flooding. One of the challenges identified through the survey was the disposal of unused or expired medicines, including antimicrobials. It was found that antimicrobials were disposed along with household garbage and there was no specialized system for collection of medicines. Therefore, solid waste management and a specialized public system for disposal of medicines is a priority for the area.

On a bivariate analysis, it was found that household size of 4 or more (*p* < 0.001) and presence of children in the household (*p* < 0.001) increased the risk of antimicrobial consumption, while poor occupational status of the head of the family (*p*- 0.026) decreased the risk. Some previous studies done in LMIC settings have also indicated that household size (42) and presence of children (43) increase antimicrobial consumption in households and various demographic groups. However other studies have yielded different results when we looked at the relationship between educational/occupational status and antimicrobial consumption. Poor educational status is usually considered as a risk factor for inappropriate use of antimicrobials (44). The difference seen in our study may have been due to access issues affecting households in which head of the family has poor occupational status. However, on a binary logistic regression model, it was found that only household size of 4 or more (p- 0.012) was significantly associated with antimicrobial consumption in the household. The association between antimicrobial use and hygiene practices were not assessed since hygiene practices were nearly universally followed and positively skewed.

The study was an attempt to assess the linkage between community-level antimicrobial use and practices related to hygiene in households in India. The systematic random sampling followed in the study can reduce the selection bias and give representative insights on the practices in the community. The data was collected by trained community health workers, and this helped to improve the rapport with the respondents since the households were already familiar with the interviewers. There were limitations to the study too. The survey was conducted after the onset of Covid19, and this may have affected the results. There are several studies showing that Covid19 had increased the overall community and personal hygiene indicators; and reduced the incidence of other upper respiratory infections (45, 46). This may have reduced the antimicrobial consumption and increased the adherence to hygiene practices in the households. The recall period and whether the patients were able to correctly differentiate antimicrobials from other medicines was also a challenge for the study, leading to a possibility of information bias.

The study showed that antimicrobial use, especially those procured over-the-counter, is low in communities with reasonable access to healthcare services and robust water, sanitation & hygiene facilities. Hygiene was a priority for the households, as shown by high adherence to several critical practices about disinfecting drinking water, safe sanitation and handwashing. Community hygiene to prevent infections should be considered an ‘entry point’ for improving the awareness about antimicrobial use and AMR. This philosophy is also reflected in India’s National Action Plan on AMR 2025–29, launched in November 2025, which calls for promoting behavior change toward sanitation and hygiene in communities through social mobilization (47). Any awareness-raising efforts on AMR or community-based intervention on AMR should have a component of infection prevention, along with key messages on appropriate use of antimicrobials. Hygiene should be an integral part of the action plans on AMR, especially those in sub-national and district settings.

## Data Availability

The raw data supporting the conclusions of this article will be made available by the authors, without undue reservation.
